# Non-Responsive Feeding Practices, Unhealthy Eating Behaviors, and Risk of Child Overweight and Obesity in Southeast Asia: A Systematic Review

**DOI:** 10.3390/ijerph14040436

**Published:** 2017-04-19

**Authors:** Ana Cristina Lindsay, Somporn Sitthisongkram, Mary L. Greaney, Sherrie F. Wallington, Praewrapee Ruengdej

**Affiliations:** 1 Department of Exercise and Health Sciences, University of Massachusetts—Boston, Boston, MA 02125, USA; 2 Department of Nutrition, Harvard School of Public Health, Boston, MA 02125, USA; 3 Department of Nursing, University of Massachusetts—Boston, Boston, MA 02125, USA; Somporn.sitthison001@umb.edu; 4 Health Studies and Department of Kinesiology, University of Rhode Island, Kingston, RI 02881, USA; mgreaney@uri.edu; 5 Lombardi Comprehensive Cancer Center, Georgetown University, Washington, DC 20057, USA; slw49@georgetown.edu; 6 Boromarajonani Nursing College Chiang Mai, Chiang Mai 50180, Thailand; praewrapeeja@gmail.com

**Keywords:** feeding practices, eating behaviors, obesity, children, parents, Southeast Asia

## Abstract

Childhood obesity is increasing dramatically in many Southeast Asian countries, and becoming a significant public health concern. This review summarizes the evidence on associations between parental feeding practices, child eating behaviors, and the risk of overweight and obesity in Southeast Asian children 2–12 years old. We systematically searched five electronic academic/research (PubMed, PsycINFO, ProQuest Nursing, Medline, and CINAHL) databases using the Preferred Reporting Items for Systematic Reviews and Meta-Analyses (PRISMA) statement for peer-reviewed studies published in English between January 2000 and December 2016. Fourteen observational studies met the inclusion criteria and were reviewed. Reviewed studies were examined separately for preschool- and school-aged children and revealed that non-responsive parental feeding practices and unhealthy child eating behaviors were associated with a risk of child overweight and obesity in several Southeast Asian countries. Nonetheless, due to the small number of identified studies (*n* = 14) and because only about half of the Southeast Asian countries (Thailand, Vietnam, Singapore, the Philippines, and Malaysia) were represented (5/11) in the examined studies, additional research is needed to further understand the factors associated with childhood obesity among children in Southeast Asia to develop interventions that are tailored to the specific needs of Southeast Asian countries and designed to address practices and behaviors that may promote childhood obesity.

## 1. Introduction

Overweight and obesity are significant global public health problems in many low- and middle-income countries [1,2]. The prevalence of overweight and obesity has increased in children in low- and middle-income countries, from 8.1% in 1980 to 12.9% in 2013 for boys and from 8.4% to 13.4% for girls [3]. This increase may be due to the children in these countries being exposed to obesogenic environments that foster excessive consumption of unhealthy foods, hinder physical activity and promote sedentary behaviors, which increase children’s risk of energy imbalance, excessive weight gain, and obesity [4,5].

Southeast (SE) Asia is a sub-region of Asia comprised of the countries south of China, east of India, west of New Guinea, and north of Australia [2]. Southeast Asia consists of Mainland Southeast Asia and Maritime Southeast Asia and includes 11 countries (Brunei, Cambodia, East Timor, Indonesia, Laos, Malaysia, Myanmar, the Philippines, Singapore, Thailand, and Vietnam) experiencing varying degrees of economic development [2,6]. The region was at the crossroads of the maritime Silk Road trade network, which resulted in cultural and religion influences from Islam, China, and India. In mainland Southeast Asia, the culture is a mix of Indochinese (Burma, Cambodia, Laos, and Thailand) and Chinese (Vietnam), while in countries like Indonesia, the Philippines, Singapore, and Malaysia the culture is a mix of indigenous Austronesian, Indian, Islamic, Western, and Chinese cultures [6]. The region has also had Western influences due to colonization by countries such as Spain (the Philippines) and Portugal (East Timor) [6]. A shared feature around the SE Asia region is rice paddy agriculture [6].

Rapid economic growth in SE Asian countries has contributed to reduced child under-nutrition rates, but also increased child over-nutrition (i.e., overweight and obesity), particularly in urban areas [2,4]. There has been an increase in the number of children who are obese in SE Asia, from 1.2 million in 1990 to 2.5 million in 2010 [1]. Moreover, wide differences exist in the prevalence of child overweight/obesity (<5 years of age) in the region. Middle-income countries like Indonesia have the highest prevalence of childhood obesity (12%), followed by Thailand (11%), Malaysia (7%), and the Philippines (5%), while the lowest prevalence rates are found in the lower-middle income countries, Myanmar (3%), Laos (2%), and Cambodia (2%) [2]. Therefore, addressing and preventing rising childhood obesity rates is a public health priority in SE Asian countries and is necessary to prevent increases in adult obesity and associated health care costs.

Children develop eating habits during childhood that have immediate and long-term consequences for their health and weight status [7]. Evidence suggests that children learn what, when, and how much to eat through family, culture, and environmental influences [7]. Parents are critical in helping their children develop and maintain healthful eating habits and food preferences [7,8]. Parental feeding practices, strategies that parents use to influence children’s eating (e.g., preferences and amounts), may be particularly important influences in the early years [9,10]. A growing body of research suggests that parental feeding practices may be associated with children developing unhealthy eating habits (e.g., consuming excessive quantities, eating in the absence of hunger, emotional eating, etc.) that increase the risk of overweight and obesity [7,9,11,12].

Cultural, socioeconomic, and psychological factors shape parents’ perceptions of and practices related to child feeding and eating behaviors [7,11]. The decision to breastfeed is the first feeding decision parents make that has long-lasting consequences for their children’s health and weight status [13]. Overall, the effects of breastfeeding on risk of child obesity are conflicting. Some studies suggest that breastfeeding confers protection against childhood overweight and obesity [13,14], and that the protective role of breastfeeding may be due to: (1) rate of weight gain in infancy, and (2) self-regulation of energy intake [13,14]. On the other hand, some studies have found that breastfeeding has little [15] or no impact [16] on children’s body mass index (BMI), and suggest that increasing breastfeeding is unlikely to reduce the global epidemic of childhood obesity.

Recent scientific evidence suggests associations between non-responsive parental feeding practices (e.g., use of overly restrictive, controlling, rewarding, or pressure feeding), unhealthy eating habits among children (e.g., disinhibited eating, lack of self-regulation of food intake, etc.) [7,9,10], and increased risk of child overweight and obesity [10]. The use of non-responsive food-related parenting feeding practices may negatively impact children’s current and future eating habits because they interfere with children’s innate internal hunger and satiety cues [7,9,10]. Specific eating habits such as skipping breakfast, excessive snacking, consumption of fast food, and/or intake of sugar-sweetened beverages (SSBs) have been linked with the risk of overweight and obesity in children [17,18,19]. Furthermore, unhealthy eating habits like under-responsiveness to internal satiety cues (low satiety responsiveness) and over-responsiveness to external food cues, such as taste, smell, availability, and emotions (high enjoyment of food, food responsiveness, and emotional overeating) are associated with an increased risk of child overweight and obesity [20,21,22].

Associations between non-responsive parental feeding practices, unhealthy child eating habits, and an increased risk of child overweight and obesity have primarily been studied in the Western context, so there is a need to determine if similar patterns occur in SE Asia. The objectives of this systematic literature review were to: (1) identify and summarize findings from existing studies examining associations between non-responsive parental feeding practices, unhealthy child eating behaviors, and risk of overweight and obesity in children aged 2–12 years living in SE Asia; (2) highlight the limitations of current studies; and (3) generate suggestions for future research.

## 2. Materials and Methods

We followed the reporting guidelines of the Preferred Reporting Items for Systematic Reviews and Meta-Analysis (PRISMA) statement when conducting this review [23]. The PRISMA statement includes guidelines that include a four-phase flow diagram to systematically guide the inclusion and exclusion of research papers. In addition, the guidelines provide a 27-item checklist that describes the requirements per review section (e.g., title, abstract, introduction, methods, results, discussions, and funding) to ensure that systematic reviews are properly conducted and reported [23].

### 2.1. Search Strategy

We searched five databases—PubMed, PsycINFO, ProQuest Nursing, Medline, and Cumulative Index to Nursing and Allied Health Literature (CINAHL). The search, conducted between August and December 2016, was limited to full-text, peer-reviewed articles published in English between January 2000 and June 2016. Search terms included: (1) child OR pediatric OR toddlers OR preschoolers; (2) weight; (3) obesity; (4) overweight; (5) feeding practices OR feeding behavior OR feeding strategy OR feeding style; (6) eating behavior OR appetitive traits OR eating habit; and (7) Southeast Asia OR Malaysia OR Singapore OR Vietnam OR Laos OR Cambodia OR Thailand OR Indonesia OR East Timor OR Philippines OR Myanmar OR Burma OR Brunei.

Two authors independently examined the titles and abstracts of all citations identified, and citations were excluded when both authors determined that the study did not meet the inclusion criteria. Next, these same two authors independently reviewed the full articles for studies that were not excluded based on title and abstracts. In addition, to identify additional potentially eligible studies, the same two authors searched the reference lists of existing full articles that satisfied the inclusion criteria. A final set of articles was agreed upon and examined to extract the relevant information pertaining to the objectives of this systematic review. The search strategy is illustrated in Figure S1 (see Supplemental Materials: PRISMA flow diagram).

### 2.2. Study Selection

This review was limited to studies that included normally developing SE Asian children (i.e., not born preterm; not diagnosed with physical or mental complications, etc.) between two and 12 years of ages (i.e., preschool and school-age children). Studies that exclusively used qualitative methods were excluded to simplify the comparison of studies’ findings. To be included in the review, studies needed to have been published in English between January 2000 and December 2016 and had to examine the association between at least one parental feeding practice and/or child eating habits and childhood overweight and/or obesity in SE Asian children aged 2–12 years.

### 2.3. Data Extraction and Data Synthesis

Two authors independently read and completed data extraction for all eligible articles using a data extraction form created to gather the following: (1) authors; (2) study setting; (3) study aim(s); (4) study population; (5) study design; (6) measure(s) of parental feeding practices and child eating habits; (7) measure(s) of child weight status (i.e., overweight and obesity); and (8) study results. The two sets of completed data extraction forms were compared, and discrepancies were discussed and resolved with feedback from a third author. Due to the range of study designs, assessment of exposure and outcomes, conducting a meta-analysis of the data was not appropriate, and results of this review are presented as a narrative summary.

### 2.4. Quality Assessment of Included Studies

Using the Strengthening in the Reporting of Observational Studies in Epidemiology (STROBE) statement [24], two authors assessed study quality, possible bias and methodological areas that may have been inadequately addressed by completing a quality checklist created for this review. The checklist consisted of 10 Yes/No questions. All *yes* answers were given a score of 1, and a score of 0 was assigned to all *no* answers for each item in the checklist (range 0–10/per paper). A score of 7 or higher was regarded as being indicative of a good-quality study. The quality assessment of included studies is presented in Table 1 (Supplementary Materials: Table S1).

## 3. Results

### 3.1. Search

**Figure 1:**
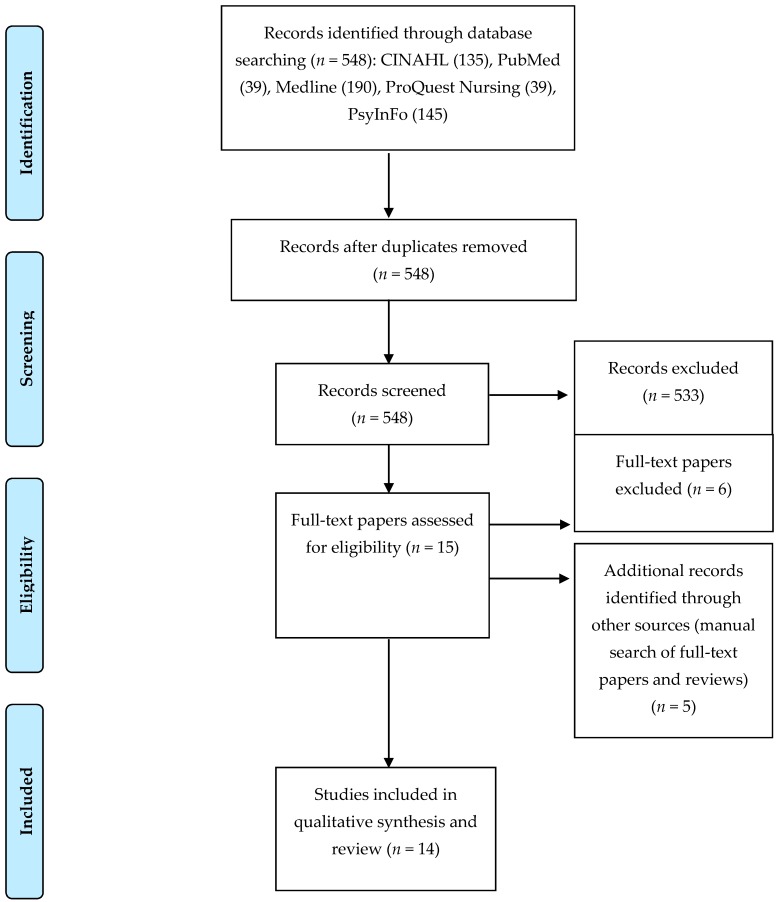
Preferred Reporting Items for Systematic Reviews and Meta-Analyses (PRISMA) flow diagram [23].

The search strategy generated 548 articles (see Figure 1). After exclusion, 14 original research articles were deemed eligible and included in this systematic review for content analysis and qualitative synthesis [25,26,27,28,29,30,31,32,33,34,35,36,37,38].

### 3.2. Summary of Included Studies

The included studies took place in five SE Asian countries (Thailand, Malaysia, Singapore, Vietnam, and the Philippines), and examined infant feeding (e.g., initiation and duration of breastfeeding), non-responsive parental feeding practices (e.g., controlling and monitoring of the child’s food intake, pressure to eat, weight-based food restriction, etc.), and a number of unhealthy child eating habits (e.g., consuming excessive quantities, drinking sugar-sweetened beverages, excessive snacking and skipping breakfast, external and internal responses to food) associated with the risk of child overweight and obesity in children living in SE Asia.

Of the 14 reviewed studies, 11 were cross-sectional [25,28,29,30,32,33,34,35,36,37,38], one was a case-control study [27], and two were longitudinal studies [26,31]. Sample sizes ranged from 99 to 1782, and children included in study samples ranged from two to 12 years old. This review is organized by age groups: (1) studies with preschool-age children (2–6 years of age, six studies); and (2) studies with school-age children (6–12 years of age, eight studies). Further details of included studies are presented in Table 2 (Supplementary Materials: Table S2: Description of studies included in systematic review), while synthesized information on methodology and main findings of the included studies is presented in Table 3 (Supplementary Materials: Table S3: Studies examining associations between feeding practices, eating behaviors and risk of overweight and obesity in children 2–12 years of age included in systematic review).

#### 3.2.1. Studies with Preschool-Age Children

Six studies examining the associations between infant feeding practices, non-responsive parental feeding practices, and/or unhealthy eating habits and the risk of child overweight and obesity in preschool-age children (2–6 years old) were included in the review [25,26,27,28,29,30].

##### Breastfeeding Initiation and Duration

Two studies [25,26] using the same cohort of preschool-age children (4–5 years) in Ho Chi Minh City, Vietnam, found a significant association between breastfeeding duration and BMI—with each additional month of breastfeeding being associated with a reduction in BMI for boys by 0.05 kg/m^2^.

##### Non-Responsive Feeding Practices

A case-control study with three- to five-year-old children in Thailand assessed both parental feeding practices and child eating behaviors using the Food Parenting Questionnaire [39] and found that the non-responsive feeding practice of low pressure to eat was associated with overweight in adjusted analyses [27]. A cross-sectional study conducted with three- to six-year old children in Vietnam [28] examined the association of non-responsive parental feeding practices (i.e., restriction, pressure to eat, and monitoring of child food intake) and child weight status (BMI) and found that the mother’s perception of the child’s weight was negatively associated with pressure to eat and positively associated with monitoring. Furthermore, a positive association was found between the mother’s perception of the child’s weight and restriction.

##### Unhealthy Eating Habits

The study conducted in Thailand [27] mentioned above that examined the relationship between child eating habits using the Child Eating Behavior Questionnaire (CEBQ) [40] and risk of child overweight determined that high food responsiveness scores (assessment of eating in response to external food cues) and high enjoyment of food scores (represents general interest in food) were associated with being overweight.

One cross-sectional study [29] conducted with a sample of three- to six-year-old children in Vietnam examined the relationship between child eating habits and risk of child overweight and obesity. Findings revealed that consuming large amounts of food and eating fast food were associated with increased risk of child overweight and obesity [29]. Similarly, a cross-sectional study conducted with four- to six-year-old children in Malaysia found that children’s body mass index had a positive relationship with fast food intake and eating out [30].

#### 3.2.2. Studies with School-Age Children

Eight studies included in this review examined associations between a number of non-responsive parental feeding practices and/or unhealthy child eating habits and the risk of child overweight and obesity in school-age children (6–12 years old) [31,32,33,34,35,36,37,38]. The examined practices and habits are discussed below.

##### Breastfeeding Initiation and Duration

Three studies examined the association between breastfeeding initiation and duration and weight status in school-age children [31,32,33]. All three found that children who were never breastfed or had been breastfed for a short duration (<4–6 months) were more likely to be classified as overweight or obese than children who were breastfed and/or were breastfed for longer durations (≥4–6 months). Importantly, after controlling for potential confounding factors, these relationships were no longer significant.

A cross-sectional study evaluating the association between breastfeeding and overweight and obesity with school-age children (10–12 years of age) conducted in Singapore found that children who were breastfed were 14% more likely to be at a healthy weight than those who were never breastfed [31]. However, after adjusting for potential confounders, this association was no longer significant.

A cross-sectional study of primary school-age children (6–10 years old) conducted in Thailand determined that, compared to children of normal weight, a higher proportion of children who were obese had not been breastfed or had only been breastfed for a short duration (<4–6 months) [32]. This association was not significant after controlling for other maternal feeding practices (control over child’s snacking, control over child’s access to high caloric food, and child characteristics (birth weight, child age)). Similarly, another cross-sectional study examining factors associated with the nutritional status of school-age children (9–10 years of age) in Malaysia found that a higher proportion of overweight and obese children were breastfed for less than six months, and that children who were of normal weight were breastfed for longer, although the association was no longer significant in the adjusted analysis [33].

##### Non-Responsive Feeding Practices

Three cross-sectional studies examined the relationship between non-responsive child feeding practices and risk of overweight and obesity in school-age children [32,34,35]. Two [34,35] of these studies were conducted in Malaysia and both used the Child Feeding Questionnaire (CFQ) by Birch et al. [41] to assess parental feeding practices. One study with children 9–12 years of age found that the maternal feeding practice of low pressure to eat was significantly associated with an increased risk of overweight [34]. Similarly, a study conducted in Malaysia with children 7–8 years of age found that parents of children who are overweight and obese had scores for pressure to eat lower than parents of normal-weight children [35]. Food restriction was positively correlated with children’s BMI, whereas pressure to eat was negatively correlated with children’s BMI [35]. Lastly, a study conducted in Thailand found that maternal control over the consumption of high caloric food and maternal control of the quantity of food intake were associated with childhood obesity [32].

##### Unhealthy Eating Habits

Four cross-sectional studies examined the relationship between unhealthy eating habits among children—including skipping breakfast, frequent fast food consumption, excess snacking, consumption of unhealthy snacks, and increased consumption of sugar-sweetened beverages—and risk of overweight and obesity in school-age children [33,36,37,38].

A cross-sectional study conducted in Malaysia with children 10–12 years of age determined that children who were overweight or obese were more likely to skip breakfast than normal-weight children [38]. Similarly, another cross-sectional study of 9–10-year-old Malaysian children found that children who were overweight and obese were more likely to consume fast food (>4 times/week) than normal-weight children [36].

A cross-sectional study of 10–12-year-old Filipino children found that overweight children ate significantly more servings of daily morning snacks than normal-weight children [37]. Children who were obese consumed significantly more servings of sugar-sweetened beverages drinks than children who were of normal weight or overweight [37].

A cross-sectional study conducted with a sample of Malaysian children aged seven to 12 years using the CEBQ [40] found that both food responsiveness (eating in response to external food cues) and desire to drink (reflecting an increased desire to drink) scores were positively associated with a child’s BMI in adjusted models, whereas satiety responsiveness, slowness in eating, and emotional under-eating subscales were negatively associated [36].

## 4. Discussion

Countries around the world are experiencing major economic transitions, urbanization, and associated lifestyle changes such as eating and physical activity patterns [1]. Changing social and physical environments are contributing to a worldwide obesity epidemic [1]. To date, a growing body of research, mostly conducted in Western countries, has demonstrated associations between breastfeeding [14,42], non-responsive parental feeding practices [10,11], children’s unhealthy eating habits [9,12,15,43,44], and increased risk of overweight and obesity in children. Therefore, this systematic review examined and summarized the evidence for these associations in children (2–12 years of age) in SE Asian countries.

Although breastfeeding may reduce children’s risk of obesity, results from the extant studies conducted in low-, middle-, and high-income countries conflict [13,14,42]. Of the five studies included in this review examining the association between breastfeeding and risk of child overweight and obesity, only one [25] found a significant association between breastfeeding practices (initiation and duration) and an increased risk of child overweight and obesity in preschool-age children after adjusting for potential confounding factors. Importantly, four [25,28,29,30] of the five studies were cross-sectional, including a study [25] that found a significant association between breastfeeding duration and child obesity, thereby preventing inferences about causality.

Mirroring a growing body of research documenting the associations between non-responsive parental feeding practices and increased risk of child overweight and obesity [9,10], findings from four studies [27,32,34,35] included in this review suggest that non-responsive parental feeding practices such as control of a child’s food consumption [32] and restriction of food intake [35] are positively associated with the risk of child overweight and obesity. On the other hand, three reviewed studies [27,34,35] found that pressure to eat was associated with the risk of child overweight. Previous studies indicate that non-responsive feeding practices interfere in the child’s natural ability to self-regulate food intake based on cues of hunger and satiety [7,9,10,11,12]. Furthermore, research suggests that both pressure to eat and eating restrictions are associated with unrestrained eating and disinhibited eating in later life, excessive weight gain, and increased risk of child obesity in children [12,45,46,47].

In agreement with prior research, five studies included in this review documented associations between children’s unhealthy eating habits (e.g., skipping breakfast, increased fast food consumption, and increased consumption of sugar-sweetened beverages) and increased risk of overweight and obesity in school-age children [27,33,36,37,38]. Previous studies have linked skipping breakfast to risk of overweight and obesity [37,38]. However, the evidence is conflicting, and the mechanism needs to be further investigated to eliminate possible confounding factors. In agreement with prior research [48,49], a cross-sectional study conducted in Malaysia [38] found that children who are overweight and obese are significantly more likely to skip breakfast than normal-weight children.

Despite the growing number of studies investigating the association between snack consumption and children’s weight status, the available evidence is inconclusive [17,50,51]. Some studies suggest that there has been an increase in the consumption of unhealthy snacks by children worldwide [17,47] and that snacks contribute a significant proportion of energy to children’s diet [17]. One study conducted in the Philippines and included in this review found that the number of snack servings a child consumes was significantly associated with child overweight [37].

In agreement with previous studies, two studies included in this review found that children who were overweight and obese consumed significantly higher amounts of SSBs than normal-weight children [33,37]. Prior research suggests that SSB intake is associated with weight status and an increased risk of child overweight and obesity [9,49]. Several available studies and reviews have suggested a direct link between consumption of SSBs and children’s weight status [9,52,53].

A growing body of literature indicates that differences in weight status may be associated with psychological sensitivity to the food environment, with obese children exhibiting higher food responsiveness, eating disinhibition, eating impulsivity, and lower satiety responsiveness [42], which may lead to an excessive energy intake, energy imbalance, and weight gain. In line with previous studies, two studies included in this review [27,36] found that food responsiveness and desire to drink scores were positively associated with a child’s weight status, whereas satiety responsiveness, slowness in eating, and emotional under-eating subscales were negatively associated.

Our evaluation of the methodologies of studies included in this systematic review suggests some limitations, and therefore caution must be taken in the interpretation of findings from this review. The majority of the included studies (11/14) employed cross-sectional study designs, which does not allow for causality to be determined. Additional longitudinal studies are necessary to understand the relationship between non-responsive feeding practices, unhealthy eating behaviors, and the risk of overweight and/or obesity in children in SE Asian countries. Furthermore, included studies examined a wide variety of child feeding practices (e.g., controlling of child food intake, restriction of foods) and unhealthy eating habits (e.g., skipping breakfast, frequent consumption of fast food and sugar-sweetened beverages), which makes it difficult to assess the comparability of the study findings. In addition, all included studies employed self-reported or administered questionnaires for the assessment of child feeding practices and eating behaviors; thus, these variables may have been misclassified. Finally, some studies included in this review did not control for potential confounders or reported limited information in the control of confounding factors in data analyses and study results. A methodological strength was the use of well-known, validated questionnaires such as the Child Feeding Questionnaire [41] and the Child Eating Behavior Questionnaire [40] by five reviewed studies [27,28,34,35,36].

Given the methodological limitations of studies included in this review, studies employing multiple methods for assessing child-feeding practices and eating behaviors, including direct observations, are needed to further examine potential associations with the increased risk of overweight and obesity in children in SE Asia. Likewise, longitudinal studies are needed to examine the associations between child feeding practices and unhealthy eating behaviors and risk of child overweight and obesity in SE Asia. Although several SE Asian countries (e.g., Thailand, Philippines, Malaysia, Vietnam, and Singapore) were represented in studies included in this review, some countries (e.g., Laos, Indonesia, East Timor, Brunei, Myanmar, and Cambodia) were not. It is probable that children in these countries are exposed to similar obesogenic food environments and changes in lifestyles as the children in countries represented in this review since this region is going through an economic transition. Nonetheless, the magnitude and context of the problem may differ among countries in SE Asia. Finally, the majority of studies (eight) included in this review focused on school-age children. The paucity of studies focusing on preschool-age children is a concern given the evidence from research of the importance of the early years in the development of children’s eating behaviors and the evidence linking unhealthy parental feeding practices and child eating behaviors to increased risk of child obesity during early childhood [7,9,11]. Future studies should consider examining these associations in preschool-age children.

Limitations of the review process should also be noted. It is possible that despite the use of a systematic guideline (i.e., PRISMA) to identify studies, we may have not identified all relevant articles due to studies being published in other formats, such as country reports, or alternative databases, or in publications in other languages not included in this review. The strengths of this review include the use of systematic criteria (e.g., PRISMA) to identify and select studies and a modified quality assessment tool for the critical appraisals of papers [23,24].

## 5. Conclusions

As childhood is a crucial period in the development of lifelong, lasting eating habits with both short- and long-term health consequences, information on factors influencing the development of healthful feeding practices and eating habits related to the risk of child overweight and obesity is needed. Several modifiable non-responsive parental feeding practices and unhealthy child eating behaviors were identified in the studies included in this review, which suggests potential targets for interventions aimed at preventing and controlling the growing childhood obesity epidemic in SE Asian countries.

Given the growing evidence that non-responsive parental feeding practices and unhealthy child eating habits are associated with an increased risk of overweight and obesity in children, further research is needed to understand these associations in SE Asian children due to the limited number of studies identified and the increasing overweight and obesity rates in children in SE Asian countries. This information will be important for the development of interventions that can change these modifiable risky practices and habits to help prevent and control the growing childhood obesity epidemic in countries in SE Asia.

## Figures and Tables

**Table 1:** Quality assessment of included studies using adapted “Strengthening the Reporting of Observational Studies in Epidemiology (STROBE)” statement.

Studies	Items										
	#1	#2	#3	#4	#5	#6	#7	#8	#9	#10	Total
Dieu et al. [25]	0	1	1	1	0	1	1	1	1	1	8
Huynh et al. [26]	1	1	1	1	0	1	1	1	0	1	8
Thongbai et al. [27]	0	1	1	1	1	1	1	0	1	0	7
Do et al. [28]	0	1	1	1	1	1	0	1	1	1	8
Do et al. [29]	0	1	1	1	1	1	0	1	1	1	8
Aziz et al. [30]	0	1	1	1	1	1	1	0	1	0	7
Sabanayagam et al. [31]	1	1	0	1	0	1	0	1	1	1	7
Yamborisut et al. [32]	0	1	0	0	1	1	0	1	1	1	6
Zaini et al. [33]	0	1	1	1	1	1	0	0	1	0	6
Serene et al. [34]	0	1	1	1	1	1	1	0	1	1	8
Wan et al. [35]	0	1	1	1	1	1	1	1	1	1	9
Tay et al. [36]	0	1	1	1	1	1	1	1	1	1	9
Gonzalez-Suarez et al. [37]	0	1	0	1	1	1	0	1	1	1	7
Soo et al. [38]	0	1	0	1	0	1	1	1	1	1	7

Note: #1. Is the study longitudinal? #2. Does the paper describe the participants’ eligibility criteria? #3. Were study participants randomly selected (or representative of the study population)? #4. Did the paper report information about the measures, including references used to assess parental feeding practices and/or children’s eating behaviors? #5. Did the study include information on instrument or scale used to assess parental feeding practices and/or child eating behaviors have acceptable reliability? #6. Did the paper report how overweight and obesity was assessed in children participating in the study? #7. Did the study provide information power calculation to detect hypothesized relationships? #8. Did the study report the number of individuals who completed each of the different measures? #9. Did the participants/respondents complete at least 80% of measures? #10. Did analyses take into account confounding factors?

**Table 2:** Description of studies included in systematic review (*n* = 14).

Characteristics	No. of Studies
**Total Number of Studies Selected**	14
**Publication Dates**	
2000–2005	1
2006–2011	7
2012–2016	6
**Study Design**	
Cohort/longitudinal	2
Case-Control	1
Cross-sectional	11
**Age Group**	
Preschool-age (2–5 years)	6
School-age (6–12 years)	8
**Southeast Asian Countries Represented**	
Malaysia	6
Thailand	2
Singapore	1
Vietnam	4
The Philippines	1
**Assessment of Parental Feeding Practices and Eating Behaviors Used**	
Children’s Eating Behavior Questionnaire (CEBQ)	2
Child Feeding Questionnaire (CFQ)	3
Other (i.e., Food Frequency Questionnaire, food record, 24-h recall)	9
**Classification of Child Overweight and Obesity Used**	
International Obesity Task Force definition	4
World Health Organization (WHO) standard	7
National standard (Thailand)	2
Asian population standard	1

**Table 3:** Studies examining the associations between parental feeding practices, child eating behaviors and risk of overweight and obesity in children 2–12 years of age included in systematic review (*n* = 14).

Author (Year) Country	Sample’s Characteristics and Study Design	Study Aim(s)	Measures of Feeding Practices and Eating Behaviors	Measures of Overweight and Obesity	Main Findings
**Preschool-Age Children**					
Dieu et al. (2007); Vietnam [25]	*n* = 670 preschool-aged children (4–5 years) in the kindergarten system: 49.6% boys, 50.4% girls. Children’s mean age = 56.2 months Cross-sectional	To assess the magnitude of overweight and obesity, and identify associated socio-demographic factors in a population of preschool-aged children in the kindergarten system of Vietnam’s largest city.	Breast-feeding was defined as being breastfed at any time. Breastfeeding duration was measured in months using an interviewer-administered, pre-coded questionnaire.	Height and weight were measured using standard methods and used to calculate child’s body mass index (BMI) using age- and sex-specific BMI cutoff points proposed by International Obesity Task Force (IOTF)	The odds of being obese significantly decreased by 5% for each additional month of breastfeeding. However, the association between breastfeeding duration was not significant after controlling for confounding factors.
Huynh et al. (2011); Vietnam [26]	*n* = 526 children aged 4–5 years in urban preschools 49% boys and 51% girls. Longitudinal	To identify risk factors associated with obesity at the community and family environment levels, and to identify individual parental and child characteristics associated with changes in adiposity indicators over a one-year period.	Interviewer-administered food frequency questionnaire (FFQ).	Height and weight were measured using standard methods and used to calculate child’s BMI using cutoff points for overweight in Asian populations (of >23 kg/m^2^)	Breast-feeding was associated with reduced risk of child obesity. The protective effect of breast-feeding appeared to be more obvious in boys than in girls (reduced BMI in boys by 0.05 units).
Thongbai et al. (2011); Thailand [27]	*n* = 615 primary caregivers of 102 overweight children (cases) and 513 normal-weight children (controls) age 3 to 5 years old. Case-control	To investigate family environmental factor as determinants of overweight among preschool children.	The Food Parenting Practices [39] were used to measure the primary caregivers’ food practices and frequency of being (a) permissive, (b) authoritarian, and (c) authoritative. The Children’s Eating Behavior Questionnaire (CEBQ) [40] to measure—food responsiveness, enjoyment of food, emotional overeating, desire to drink, satiety responsiveness, slowness in eating, emotional under-eating, and food fussiness.	Height and weight were measured using the standard methods. Child nutritional status was classified by using a Ministry of Public Health growth reference for two- to seven-year-old Thais.	Three maternal feeding practices were associated with child overweight (low pressure, low encouragement through material reward, and low negotiation) without adjusting for confounding factors. Low maternal pressure was associated with child overweight adjusting for confounding factors.
Do et al. (2015); Vietnam [28]	*n =* 2677 children (rural 1313; urban 1364), aged 3–6 years. Cross-sectional	To describe the use of parental non-responsive feeding practices (i.e., restriction, pressure to eat, and monitoring of child food intake). To identify associations between the parental feeding practices and children’s diet and BMI.	The Child Feeding Questionnaire (CFQ) [41] was used to assess parental attitudes, beliefs and practices related to feeding children. Children’s diet question was used to assess children’s level of food consumption including (1) amount of food and (2) fatty food, sweets and snacks consumption.	Height and weight were measured using standard methods and used to calculate child’s BMI.	Child’s BMI and the mother’s perception of the child’s weight was negatively associated with pressure to eat and positively associated with monitoring. Restriction was positively associated with mother’s perception of the child’s weight. High consumption of fatty foods, sweets, and snacks was associated with high restriction and monitoring in rural areas and high restriction and pressure to eat but low monitoring in urban areas. The amount of food consumed was negatively associated with pressure to eat in rural areas, but positively associated with monitoring in urban areas.
Do et al. (2015); Vietnam [29]	*n* = 2677 children (rural 1313; urban 1364), aged 3–6 years. Cross-sectional	To estimate prevalence of overweight and obesity for preschool children in both urban and rural areas and to identify risk factors of overweight and obesity among children.	Structured questionnaires included amount of food, food consumption, fast eating, irregular snacks, outdoor physical activity, indoor physical activity, sedentary time, family economy, mother’s education, household size, watching food advertisements, and snack availability. These questionnaires were used to assess child eating habits and lifestyle by interviewing parents or caregivers.	Height and weight were measured using standard methods and used to calculate child’s BMI. Overweight and obesity were defined using World Health Organization (WHO) standards.	In urban areas, overweight or obesity in children were significantly associated with age, large amounts of food, fast eating, and indoor activity. In rural areas, overweight or obesity in children was significantly associated with age, frequent consumption of fatty, and mothers watching food advertisements on television (TV).
Aziz et al. (2012); Malaysia [30]	*n* = 142 children (urban 100; rural 42): aged 4–6 years, 45.8%; 52.8% boys and 47.2% girls. Cross-sectional	To compare and investigate the relationship between the nutritional status and eating practice.	Parental feeding practices questions were used to measure parental feeding practices. Children’s dietary habit questions and the three-day diet record were used to assess children’s diet intake.	Height and weight were measured using standard methods and used to calculate child’s BMI, which was used to determine weight status using the WHO and Centers for Disease Control and Prevention (CDC) 2000 growth chart as a reference.	There were significant differences in nutrient intake between children in rural and urban areas. Children’s BMI had a positive relationship with fast food intake (*r* = 0.274, *p* < 0.05) and eating out (*r* = 0.207, *p* < 0.05)
**School-Age Children**					
Sabanayagam et al. (2009); Singapore [31]	*n* = 797 school children aged 10–12; 49% girls and 51% boys. Longitudinal	To evaluate the association between breastfeeding and overweight and obesity.	Parents completed an interviewer- administered survey that assessed whether the participating child was ever breast-fed, duration of breastfeeding and type of breastfeeding. Breastfeeding was dichotomized (yes/no). Breastfeeding duration was categorized into two groups (≤3 months, >3 months). Type of breastfeeding was defined as exclusive (fed no food other than breast milk); mostly (breast milk and non-formula supplements such as water, sweetened water or juices); or partly (breast milk supplemented with formula milk or other complementary foods).	Height and weight were measured using standard methods and used to calculate child’s BMI using the IOTF cutoff points to determine weight status.	There were no significant associations between breastfeeding status (yes/no), duration of breastfeeding ≤3 months, >3 months) and type of breastfeeding and overweight and/or obesity for the cohort and by sex.
Yamborisut et al. (2006); Thailand [32]	*n* = 199 children aged 6–10. Mean age was 8.2 years. Cross-sectional	To examine the influence of family characteristics and maternal feeding practices on eating behaviors, food consumption, and nutritional status of children.	Used an FFQ and 24-h food recall to assess the food consumption patterns of the children for two days.	Height and weight were measured using standard methods and used to classify child’s nutritional status using weight-for-height Z score (WHZ). The Thai growth reference was used to classify weight status.	Maternal control over the consumption of high caloric food and large amounts of food consumed by the children was associated with childhood obesity. Compared to normal-weight children, a higher proportion of obese children were not breastfed.
Zaini et al. (2005); Malaysia [33]	*n* = 1405 students aged 9–10 years old. Mean age 9.68 years old. Cross-sectional	To examine factors affecting the nutritional status of the sample.	Questionnaire administered to students, with confirmation by mothers whenever possible. Dietary practices assessed included having breakfast, regularity of having the three main meals, types, and quantities of each food item consumed during each meal in a typical day, and the frequency of eating fast food.	Height and weight were measured using standard methods and used to calculate child’s BMI; the IOTF standard was used to classify weight status.	There was association between the proportion of students who were breastfed for more than six months and classified as overweight and obese and the proportion of students who were breastfed for less than six months and classified as overweight and obese (21.9% vs. 20.4%). Students who consumed fast food more 4+/week were more likely to be overweight/obese (24%) than those who consumed fast food less <4/week (20%).
Serene et al. (2011); Malaysia [34]	*n* = 1430 Children ages 9–12 years. Mean age for children was 10.3+/−0.8 years. Cross-sectional	To explore the association between familial and socio-environmental factors and childhood obesity.	The CFQ [41] was used to monitor parental feeding strategies and ideas (perceived parent weight, perceived child weight, perceived responsibility, concern about child’s weight, and restriction and pressure to eat).	Height and weight were measured using standard methods and used to calculate the child’s BMI, which was used to determine the weight status using the WHO growth reference for children aged 5–19 years of age.	Pressure to eat showed a reverse association with child’s risk of overweight and obesity.
Wan et al. (2012); Malaysia [35]	*n* = 175 children aged 7–8 years old. Mean age of children was 7.4+/−0.5 years. Cross-sectional	To examine the association between parental children feeding practices and child’s weight status.	Used the CFQ [41] to measure several practices including perceived weight status, food restriction and pressure to eat.	Height and weight were measured using standard methods and used to calculate child’s BMI using the BMI-for-age WHO Growth chart as standard reference.	Perception of child’s weight, perceived parental weight, and food restriction factors were positively associated with the child’s BMI, whereas pressure to eat was negatively associated with child’s BMI.
Tay et al. (2016); Malaysia [36]	*n* = 1782 children aged 7–12 years old; 48.6% boys and 51.4% girls. Cross-sectional	To determine the association between eating behaviors and BMI, BMI-for-age Z-score (BAZ), waist circumference (WC), and percentage body fat (%BF) as indicators of nutritional status and body composition the sample.	The CEBQ [40] to measure - food responsiveness, enjoyment of food, emotional overeating, desire to drink, satiety responsiveness, slowness in eating, emotional under-eating, and food fussiness.	Height and weight were measured using standard methods and used to calculate the child’s BMI using the WHO growth standards. WC was measured using standard methods. %BF was measured by bioelectrical impedance.	Food responsiveness was positively associated with body adiposity for both sexes. Desire to drink was positively associated with BMI and WC for girls. Satiety responsiveness was negatively associated with body adiposity in both sexes except for %BF of boys. Slowness in eating was negatively associated with WC in girls. Emotional under-eating was negatively associated with the body adiposity (BMI) of boys.
Gonzalez-Suarez et al. (2015); The Philippines [37]	*n* = 396 elementary school students aged 10–12 years old. Cross-sectional.	To assess the associations between snacking (e.g., time, frequency, amount, type of snacks) and risk of overweight and obesity.	Data about mid-morning, mid-afternoon (both at school during the week), and nighttime snacks were collected using an interviewer-administered 24-h food recall.	Height and weight were measured using standard methods and used to calculate the child’s BMI. The IOTF’s gender- and age-specific cutoff points were used to determine weight status.	The odds of being overweight were associated with high total snack servings of 2.12. The odds of being obese (both males and females) were associated with calories obtained from snacking.
Soo et al. (2011); Malaysia [38]	*n* = 278 urban Chinese primary school children aged 10–12; 51.8% boys and 48.2% girls. Cross-sectional	To assess the relationship between nutritional statuses and dietary habits among sample.	Dietary habits were assessed using a three-day food record. Children were asked to record the type of foods, quantity, and portion size of all food consumed.	Height and weight were measured using standard methods and used to calculate the child’s BMI, which was used to determine the weight status using the WHO BMI-for-age growth chart as a reference.	Children classified as obese skipped breakfast more frequently than those classified as having a normal weight group.

## Supplementary Materials

The following are available online at www.mdpi.com/1660-4601/14/4/436/s1, Figure S1: PRISMA flow diagram, Table S1: Quality assessment of included studies using adapted “Strengthening the Reporting of Observational Studies in Epidemiology (STROBE)” statement, Table S2: Description of studies included in systematic review, Table S3: Studies examining associations between feeding practices, eating behaviors and risk of overweight and obesity in children 2–12 years of age included in systematic review.
