# Supplementary material for: Non-Responsive Feeding Practices, Unhealthy Eating Behaviors, and Risk of Child Overweight and Obesity in Southeast Asia: A Systematic Review
Source: Int J Environ Res Public Health. 2017 Apr 19;14(4):436. doi: 10.3390/ijerph14040436 (PMC5409637; doi:10.3390/ijerph14040436)
Supplement: Supplementary file 1 [file ijerph-14-00436-s001.pdf]

Figure S1: PRISMA 2009 flow diagram*

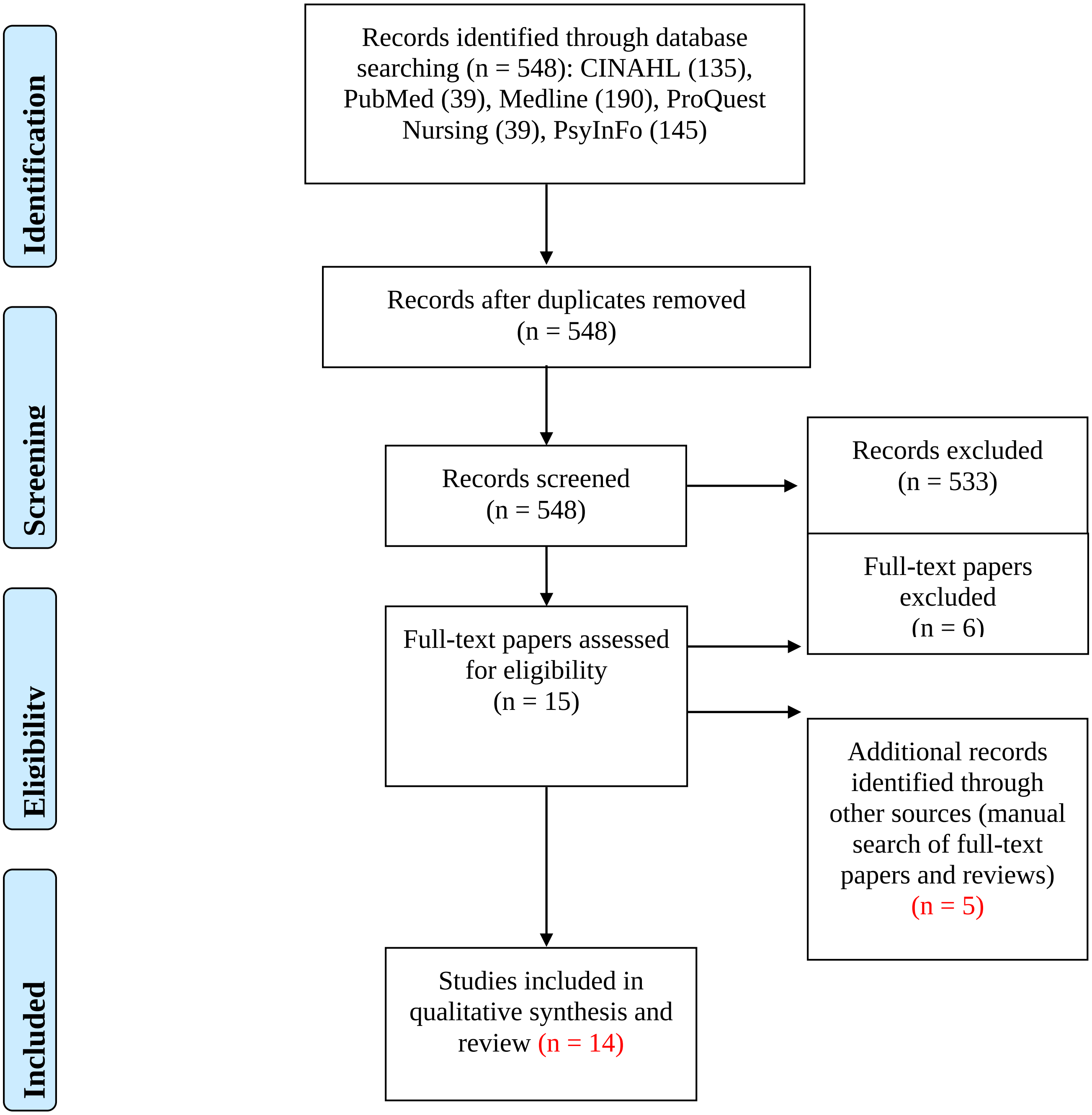

**From:* Moher D, Liberati A, Tetzlaff J, Altman DG, The PRISMA Group (2009). *P*referred *R*eporting *I*tems for *S*ystematic Reviews and *M*eta-*A*nalyses: The PRISMA Statement. PLoS Med 6(7): e1000097. doi:10.1371/journal.pmed1000097

Table S1. Quality assessment of included studies using adapted "Strengthening the Reporting of Observational Studies in Epidemiology (STROBE)" statement.

Items:

#1. Is the study a longitudinal?
#2. Does the paper describe the participants' eligibility criteria?
#3. Were study participants randomly selected (or representative of the study population)?
#4. Did the paper report information about the measures, including references used to assess parental feeding practices and/or children's eating behaviors?
#5. Did the study include information on instrument or scale used to assess parental feeding practices and/or child eating behaviors have acceptable reliability?
#6. Did the paper report how overweight and obesity was assessed in children participating in the study?
#7. Did the study provide information power calculation to detect hypothesized relationships?
#8. Did the study report the number of individuals who completed each of the different measures?
#9. Did the participants/respondents complete at least 80% of measures?
#10. Did analyses take into account confounding factors?

	Items										
**Studies**	**#1**	**#2**	**#3**	**#4**	**#5**	**#6**	**#7**	**#8**	**#9**	**#10**	**Total**
Dieu et al. [25]	0	1	1	1	0	1	1	1	1	1	8
Huynh et al. [26]	1	1	1	1	0	1	1	1	0	1	8
Thongbai et al. [27]	0	1	1	1	1	1	1	0	1	0	7
Do et al. [28]	0	1	1	1	1	1	0	1	1	1	8
Do et al. [29]	0	1	1	1	1	1	0	1	1	1	8
Aziz et al. [30]	0	1	1	1	1	1	0	1	1	0	7
Sabanayagam et al. [28]	1	1	0	1	0	1	0	1	1	1	7
Yamborisut et al. [29]	0	1	0	0	1	1	0	1	1	1	6
Zaini et al. [30]	0	1	1	1	1	1	0	0	1	0	6
Serene et al. [31]	0	1	1	1	1	1	1	0	1	1	8
Wan et al. [32]	0	1	1	1	1	1	1	1	1	1	9
Tay et al. [33]	0	1	1	1	1	1	1	1	1	1	9
Gonzalez-Suarez et al. [34]	0	1	0	1	1	1	0	1	1	1	7
Soo et al. [35]	0	1	0	1	0	1	1	1	1	0	7

Table S2. Description of studies included in systematic review (n = 14)

Characteristics	No. of Studies
**Total number of studies selected**	14
**Publication dates**	
2000 – 2005	1
2006 – 2011	6
2012 – 2016	6
**Study design**	
Cohort/longitudinal	2
Case-Control	1
Cross-sectional	11
**Age group**	
Preschool-age (2 – 5 years)	6
School-age (6 – 12 years)	8
**Southeast Asian countries represented**	
Malaysia	6
Thailand	2
Singapore	1
Vietnam	4
The Philippines	1
**Assessment of parental feeding practices and eating behaviors used**	
Children's Eating Behavior Questionnaire (CEBQ)	2
Child Feeding Questionnaire (CFQ)	3
Other (i.e., Food Frequency Questionnaire, Food record, 24-hour recall)	9
**Classification of child overweight and obesity used**	
International Obesity Task Force definition	4
World Health Organization (WHO) standard	7
National standard (Thailand)	2
Asian population standard	1

Table S3. Studies examining associations between feeding practices, eating behaviors and risk of overweight and obesity in children 2-12 years of age included in systematic review (n = 14).

Author (Year) Country	Sample's characteristics and study design	Study Aim (s)	Measures of feeding practices and eating behaviors	Measures of overweight and obesity	Main Findings
**Preschool-age Children**					
Dieu et al. (2007) Vietnam	*n* = 670 preschool-aged children (4-5 years) in the kindergarten system: 49.6% boys, 50.4% girls. Children's Mean age =56.2 months. Cross-sectional	To assess the magnitude of overweight and obesity, and identify associated socio-demographic factors in a population of preschool-aged children in the kindergarten system of Vietnam's largest city	Breast-feeding was defined as being breastfed at any time. Breastfeeding duration was measured in months using an interviewer-administered, pre-coded questionnaire.	Height and weight measured using standard methods and used to calculate child's BMI using age- and sex-specific BMI cut-off points proposed by International Obesity Task Force (IOTF)	The odds of being obese significantly decreased by 5% for each additional month of breastfeeding. However, the association between breastfeeding duration was not significant after controlling for confounding factors.
Huynh et al. (2011) Vietnam	*n* = 526 children aged 4–5 years in urban preschools 49% boys and 51% girls. Longitudinal	To identify risk factors associated with obesity at the community and family environment levels, and to identify individual parental and child characteristics associated with changes in adiposity indicators over a one-year period.	Interviewer-administered food frequency questionnaire (FFQ).	Height and weight measured using standard methods and used to calculate child's BMI using cut-off points for overweight in Asian populations (of >23 kg/m^2)	Breast-feeding was associated with reduced risk of child obesity. The protective effect of breast-feeding appeared to be more obvious in boys than in girls (reduced BMI in boys by 0.05 units).

Author (Year) Country	Sample's characteristics and study design	Study Aim (s)	Measures of feeding practices and eating behaviors	Measures of overweight and obesity	Main Findings
**Preschool-age Children**					
Thongbai et al. (2011) Thailand	*n* = 615 primary caregivers of 102 overweight children (cases) and 513 normal weight children (controls) age 3 to 5 years old. Case-control	To investigate family environmental factor as determinants of overweight among preschool children.	The Food Parenting Practices [53] was used to measure the primary caregivers' food practices and frequency of being (a) permissive, (b) authoritarian, and (c) authoritative. The Children's Eating Behavior Questionnaire (CEBQ) [52] to measure - food responsiveness, enjoyment of food, emotional overeating, desire to drink, satiety responsiveness, slowness in eating, emotional under-eating, and food fussiness.	Height and weight measured using the standard methods. Child nutritional status was classified by using a Ministry of Public Health growth reference for 2- to 7-year-old Thais.	Three maternal feeding practices were associated with child overweight (low pressure, low encouragement through material reward, and low negotiation) without adjusting for confounding factors. Low maternal pressure was associated with child overweight adjusting for confounding factors.
Aziz et al. (2012) Malaysia	n = 142 children (urban 100; rural 42): aged 4-6 years, 45.8% of children was 6 years old. 52.8% boys, 47.2% girls. Cross-sectional	To compare and investigate the relationship between the nutritional status and eating practice.	Parental feeding practices questions were used to measure parental feeding practices. Children dietary habit questions and the three days diet record were used to assess children's diet intake.	Height and weight measured using standard methods and used to calculate child's BMI, which was used to determine weight status using the WHO and CDC 2000 growth chart as a reference.	There were significant differences in nutrient intake between children in rural and urban areas. Children's BMI had a positive relationship with fast food intake ($r = 0.274$, $p = < 0.05$) and eating out ($r = 0.207$, $p < 0.05$)

Author (Year) Country	Sample's characteristics and study design	Study Aim (s)	Measures of feeding practices and eating behaviors	Measures of overweight and obesity	Main Findings
**Preschool-age Children**					
Do et al. (2015) Vietnam	*n* = 2677 children (rural 1313; urban 1364), aged 3-6 years.<br>Cross-sectional	To describe the use of parental non-responsive feeding practices (i.e., restriction, pressure to eat, and monitoring of child food intake).<br>To identify associations between the parental feeding practices and children's diet and BMI.<br>.	The Child Feeding Questionnaire (CFQ) [51] was used to assess parental attitudes, beliefs and practices related to feeding children. Children's diet question was used to assess children's level of food consumption including 1) amount of food and 2) fatty food, sweets and snacks consumption.	Height and weight measured using standard methods and used to calculate child's BMI.	Child's BMI and the mother's perception of the child's weight was negatively associated with pressure to eat and positively associated with monitoring. Restriction was positively associated with mother's perception of the child's weight. High consumption of fatty food, sweets and snacks was associated with high restriction and monitoring in rural area and high restriction and pressure to eat but low monitoring in urban area. The amount of food consumed was negatively associated with pressure to eat in rural area, but positively associated with monitoring in urban area.
Do et al. (2015) Vietnam	n = 2677 children (rural 1313; urban 1364), aged 3-6 years.<br>Cross-sectional	To estimate prevalence of overweight and obesity for preschool children in both urban and rural areas and to identify risk factors of overweight and obesity among children.	Structured questionnaires included amount of food, food consumption, fast eating, irregular snacks, outdoor physical activity, indoor physical activity, sedentary time, family economy, mother's education, household size, watching food advertisements, and snack availability. These questionnaires were used to assess child eating habits and lifestyle by interviewing parents or caregivers.	Height and weight measured using standard methods and used to calculate child's BMI.<br>Overweight and obesity were defined using WHO standards.	In the urban area, overweight or obesity in children was significant associations with age, large amounts of food, fast eating and indoor activity. In rural area, overweight or obesity in children was significant associations with age, frequent consumption of fatty, and mothers watching food advertisements on TV.

**Author (Year) Country**	**Sample's characteristics and study design**	**Study Aim (s)**	**Measures of feeding practices and eating behaviors**	**Measures of overweight and obesity**	**Main Findings**
**School-age Children**					
Zaini et al. (2005) Malaysia	*n* = 1,405 students aged 9–10 years old. Mean age 9.68 years old. Cross-sectional	To examine factors affecting the nutritional status of the sample.	Questionnaire administered to students with confirmation by mothers whenever possible. Dietary practices assessed included having breakfast, regularity of having the three main meals, types and quantities of each food item consumed during each meal in a typical day, and frequency of eating fast food.	Height and weight was measured using standard methods and used to calculate child's BMI and the IOTF standard was used to classify weight status.	There was association between the proportion of students who were breastfed for more than six months and classified as overweight and obese and the proportion of students who were breastfed for less than six months and classified as overweight and obese (21.9% vs. 20.4%). Students who consumed fast food more 4+/week were more likely to be overweight/obese (24%) than those who consumed fast food less <4/week (20%).
Yamborisut et al. (2006) Thailand	*n* = 199 children aged 6 to 10 Children mean age was 8.2 years. Cross-sectional	To examine the influence of family characteristics and maternal feeding practices on eating behaviors, food consumption, and nutritional status of children.	Used an FFQ and 24-hour food recall to assess the food consumption patterns of the children for two days.	Height and weight measured using standard methods and used to classify child's nutritional status using weight-for-height Z score (WHZ). The Thai growth reference was used to classify weight status.	Maternal control over the consumption of high caloric food and large amounts of food consumed by the children was associated with childhood obesity. Compared to normal weight children, a higher proportion of obese children were not breastfed.

Author (Year) Country	Sample's characteristics and study design	Study Aim (s)	Measures of feeding practices and eating behaviors	Measures of overweight and obesity	Main Findings
**School-age Children**					
Sabanayagam et al. (2009) Singapore	*n* = 797 school children aged 10-12. 49% girls and 51% boys. Longitudinal	To evaluate the association between breastfeeding and overweight and obesity.	Parents completed an interviewer- administered survey that assessed whether the participating child was ever breast-fed, duration of breastfeeding and type of breast-feeding. Breast-feeding was dichotomized (yes/no). Breastfeeding duration was categorized into two groups (≤3 months, >3 months). Type of breast-feeding was defined as exclusive (fed no food other than breast milk); mostly (breast milk and non-formula supplements such as water, sweetened water or juices); or partly (breast milk supplemented with formula milk or other complementary foods).	Height and weight measured using standard methods and used to calculate child's BMI using the IOTF cutoff points to determine weight status.	There were no significant associations between breastfeeding status (yes/no), duration of breastfeeding 3 months, >3 months) and type of breastfeeding and overweight and/or obesity for the cohort and by sex.
Serene et al. (2011) Malaysia	*n* = 1430 Children ages 9 – 12 years. Mean age for children was 10.3+/−0.8 years. Cross-sectional	To explore the association between familial and socio-environmental factors and childhood obesity.	The Child Feeding Questionnaire (CFQ) [51] was used to parental feeding strategies, and ideas (perceived parent weight, perceived child weight, perceived responsibility, concern about child's weight, and restriction and pressure to eat).	Height and weight measured using standard methods and used to calculate child's BMI, which was used to determine weight status using the WHO growth reference for children aged 5–19 years of age.	Pressure to eat showed a reverse association with child's risk of overweight and obesity.

Author (Year) Country	Sample's characteristics and study design	Study Aim (s)	Measures of feeding practices and eating behaviors	Measures of overweight and obesity	Main Findings
**School-age Children**					
Soo et al. (2011) Malaysia	*n* = 278 urban Chinese primary school children aged 10 to 12 51.8% boys and 48.2% girls. Cross-sectional	To assess the relationship between nutritional status, dietary habits among sample.	Dietary habits were assessed using a 3-day food record. Children were asked to record the type of foods, quantity, and portion size of all food consumed.	Height and weight measured using standard methods and used to calculate child's BMI, which was used to determine weight status using the WHO BMI-for-age Growth chart as a reference.	Children classified as obese skipped breakfast more frequently than those classified as having a normal weight group.
Wan et al. (2012) Malaysia	*n* = 175 children ages 7-8 years old. Mean age of children was 7.4+/−0.5 years. Cross-sectional	To examine the association between parental child feeding practices and child's weight status.	Used the Child Feeding Questionnaire to measure several practices including perceived weight status, food restriction and pressure to eat [51].	Height and weight measured using standard methods was used to calculate child's BMI using the BMI-for-age WHO Growth chart as standard reference.	Perception of child's weight, perceived parental weight, and food restriction factors were positively associated with child's BMI, whereas pressure to eat was negatively associated with child's BMI.
Gonzalez-Suarez et al. (2015) The Philippines	*n* = 396 elementary school students age 10–12 years old, Cross-sectional.	To assess the associations between snacking (e.g. time, frequency, amount, type of snacks) and risk of overweight and obesity.	Data about—mid-morning, mid-afternoon (both at school during the week), and nighttime snack was collected using an interviewer-administered 24-hour food recall.	Height and weight measured using standard methods and used to calculate child's BMI using the (IOTF's gender-and age-specific cutoff points was used to determine weight status.	The odds of being overweight was associated with high total snack servings was 2.12. The odds of being obese (both males and females) were associated with calories obtained from snacking.

**Author (Year) Country**	**Sample's characteristics and study design**	**Study Aim (s)**	**Measures of feeding practices and eating behaviors**	**Measures of overweight and obesity**	**Main Findings**
**School-age Children**					
Tay et al. (2016) Malaysia	*n* = 1782 of children age 7 to 12 years old. 48.6% boys and 51.4% girls. Cross-sectional	To determine the association between eating behaviors and BMI, BMI-for-age Z-score (BAZ), waist circumference (WC), and percentage body fat (%BF) as indicators of nutritional status and body composition the sample.	The Children's Eating Behavior Questionnaire (CEBQ) [52] to measure - food responsiveness, enjoyment of food, emotional overeating, desire to drink, satiety responsiveness, slowness in eating, emotional under-eating, and food fussiness.	Height and weight measured using standard methods and used to calculate child's BMI using the WHO growth standards. WC was measured using standard methods. %BF was measured by bioelectrical impedance.	Food responsiveness was positively associated with body adiposity for both sexes. Desire to drink was positively associated with BMI and WC for girls. Satiety responsiveness was negatively associated with body adiposity in both sexes except for %BF of boys. Slowness in eating was negatively associated with WC in girls. Emotional under-eating was negatively associated with the body adiposity (BMI) of boys.
